# Collective cancer cell invasion induced by coordinated contractile stresses

**DOI:** 10.18632/oncotarget.5874

**Published:** 2015-10-30

**Authors:** Angela M. Jimenez Valencia, Pei-Hsun Wu, Osman N. Yogurtcu, Pranay Rao, Josh DiGiacomo, Inês Godet, Lijuan He, Meng-Horng Lee, Daniele Gilkes, Sean X. Sun, Denis Wirtz

**Affiliations:** ^1^ Department of Chemical and Biomolecular Engineering, The Johns Hopkins University, Baltimore, Maryland, 21218, USA; ^2^ Physical Sciences-Oncology Center and Institute for NanoBioTechnology, The Johns Hopkins University, Baltimore, Maryland, 21218, USA; ^3^ Department of Mechanical Engineering, The Johns Hopkins University, Baltimore, Maryland, 21218, USA; ^4^ Department of Oncology and Department of Pathology, The Johns Hopkins School of Medicine, Baltimore, Maryland, 21218, USA

**Keywords:** spatio-temporal invasion, fibrosarcoma, 3D invasion model

## Abstract

The physical underpinnings of fibrosarcoma cell dissemination from a tumor in a surrounding collagen-rich matrix are poorly understood. Here we show that a tumor spheroid embedded in a 3D collagen matrix exerts large contractile forces on the matrix before invasion. Cell invasion is accompanied by complex spatially and temporally dependent patterns of cell migration within and at the surface of the spheroids that are fundamentally different from migratory patterns of individual fibrosarcoma cells homogeneously distributed in the same type of matrix. Cells display a continuous transition from a round morphology at the spheroid core, to highly aligned elongated morphology at the spheroid periphery, which depends on both β_1_-integrin-based cell-matrix adhesion and myosin II/ROCK-based cell contractility. This isotropic-to-anisotropic transition corresponds to a shift in migration, from a slow and unpolarized movement at the core, to a fast, polarized and persistent one at the periphery. Our results also show that the ensuing collective invasion of fibrosarcoma cells is induced by anisotropic contractile stresses exerted on the surrounding matrix.

## INTRODUCTION

Approximately 40% of the 200,000 patients diagnosed with sarcoma worldwide will succumb from metastatic disease [1, 2]. Fibrosarcoma is a subtype of soft tissue sarcomas [3]. Metastasis remains the most common cause of fibrosarcoma-associated deaths [4]; the survival rate for high-grade tumors is only 30% [5]. Clinical characteristics of fibrosarcoma are poorly understood and described [4]. One of the major challenges in treating fibrosarcoma is the difficulty in determining the surgical resection area due to the infiltrative nature of the cells, which results in high (>50%) recurrence rate. Thus, understanding the process of fibrosarcoma cell dissemination is critical to developing new strategies to treat this disease.

The spheroid tumor system has been extensively used as an *in vitro* model to recapitulate the *in vivo* tumor microenvironment and study the initial steps of invasion from a primary tumor [6–8]. This assay consists of embedding multicellular spheroids inside three-dimensional (3D) extracellular matrices (ECM) such as collagen I, which allows for both cell-cell and cell-ECM interactions. This 3D invasion model has been previously utilized to investigate the molecular mechanisms that govern angiogenic sprouting of endothelial spheroids inside collagen gels [9] and the role of MMPs in cancer cell invasion [7]. Recent studies have shown that human fibrosarcoma cells that are well-dispersed in a matrix adopt fundamentally different strategies for migration from cells migrating on 2D substrates [10]. However, it is unclear how fibrosarcoma cells within the tumor spheroid – in which cells have close cell-cell contacts with their neighbors – may “prepare” cells at the spheroid periphery to present the correct morphology and polarization for effective invasion into the surrounding matrix.

Here, we developed and analyzed a 3D encapsulated spheroid-matrix system to investigate cancer cell invasion into an adjacent collagen matrix. We perfomed dynamic single-cell resolution measurements and report the spatial and temporal kinetics in the morphology and motility behavior of individual cells inside the spheroids. Using this model system, we characterize the invasion profiles of spheroids and identify the role of cell contractility and cell-matrix interactions as essential mediators of cancer cell invasion. We also show that cell invasion in the surrounding matrix requires a large net contractile force exerted by the spheroid on its surroundings before invasion can occur. In addition, cells move persistently toward the invasive front of the spheroid and this behavior is fundamentally different from a homogeneously distributed population of single cells embedded inside similar 3D gels.

## RESULTS

### Fibrosarcoma cell invasion and spreading from a spheroid in 3D collagen matrix

**Figure 1:**
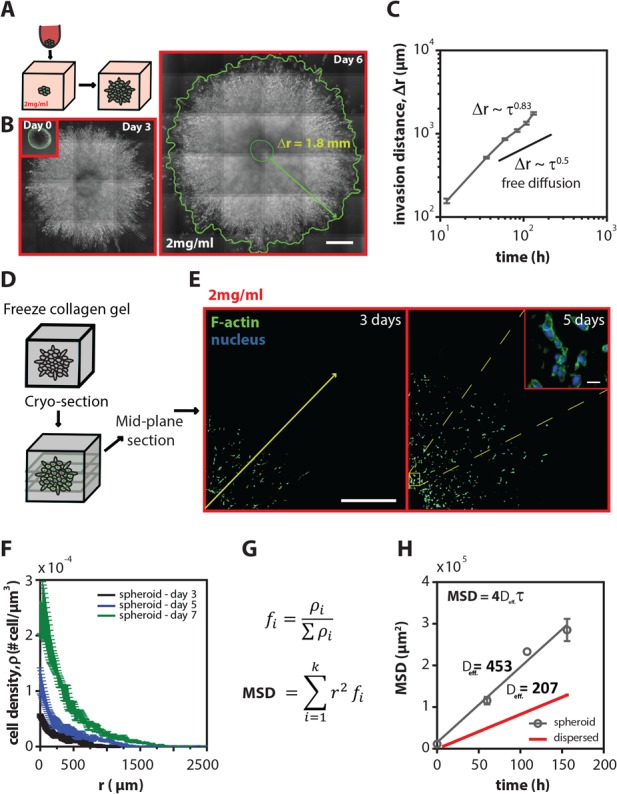
Tumor spheroids are highly invasive inside 3D collagen matrices **A.** Schematic of the experimental procedure: tumor spheroid preparation, embedding, and invasion inside a 3D collagen matrix. **B.** Representative stitched phase-contrast images showing human fibrosarcoma HT1080 spheroids at 0, 3, and 6 days after embedding inside 2 mg/ml collagen matrices. The spheroid growing front was manually traced to obtain invasion distance, Δr. Scale bars, 500 μm **C.** Mean time-dependent invasion distance ± SEM of HT1080 spheroids as a function of time. The invasion distance of HT1080 tumor spheroids was determined by measuring the distance between spheroid-matrix interface and the spheroid initial radius (Δ*r* = *r*(*t*) - *r*(0)). The exponent of invasion distance profiles has a value of 0.83, indicating that spheroid invasion in 3D matrix is a highly invasive process. An exponent of 0.5 is indicative of free diffusion. **D.** Schematic of the frozen collagen containing tumor spheroid prior to cryo-sectioning **E.** Representative stitched fluorescent images of the mid-plane cryo-stat section (thickness, 10 μm) of spheroids grown inside 2 mg/ml collagen gels for 3 and 5 days (blue-nuclei and green-actin filaments). Inset shows the dispersion of the cells in the middle of the spheroid. Scale bars represent 500 μm and 20 μm in the inset. **F.** Mean cell density ± SEM as a function of radius measured from the mid-plane cryo-sections at 3, 5, and 7 days. **G.** Equation used to approximate the MSD of cells inside spheroids taking into account the local cell density. **H.** Mean square displacement (MSD) ± SEM for cells grown in tumor spheroids as well as for cells homogeneously distributed inside 3D matrices (dispersed). The MSD of a population of homogeneously distributed single cells was calculated as explained in the materials and method section.

Collagen I is by far the most abundant component of human connective tissues and is also the main component of the additional extracellular matrix deposited by carcinoma and sarcoma tumors in their periphery [11–13]. Here we focus on fibrosarcoma, a malignant metastatic tumor of fibrous connective tissues [14] using HT1080 human fibroscarcoma, a cell line used extensively in cell invasion and migration studies [10, 15–19]. To study fibrosarcoma invasion and growth in 3D microenvironments, cell spheroids—average initial radius of 174 μm—were embedded inside 3D collagen I matrices (Figure 1A and Supplementary Figure S2A).

The invasion (or spreading) distance of HT1080 tumor spheroids was determined by measuring the distance between spheroid-matrix interface and the spheroid initial radius (Δ*r* = *r*(*t*)*–r*(0)), and showed a > 10 fold increase from its initial value over 6 days (Figure 1B). To characterize the time-dependent growth of the spheroids and the mode of invasion of the cells into the surrounding collagen matrix, the spheroid was continuously monitored for 7 days (Figure 1C). If the invasion resulted from persistent random-walk migration of individual cancer cells in the spheroids; then, the invasion distance would increase with time at a rate of Δ*r* ~ *t*^1/2^ [20, 21] at long time scale. However, our results showed that the invasion distance of fibrosarcoma cells propagated over time at a rate of Δ*r* ~ τ^β^ with an exponent of invasion *β* = 0.83 ± 0.06. This result suggested that cell spheroids were highly invasive and that this invasion process was fundamentally different from the case of homogeneously distributed (individual) cells embedded in 3D collagen gels at low density which undergo the so-called anisotropic random-walk migration [10] and from the case of cohesively growing spheroids which switch from exponential to linear growth beyond the crossover region (200 μm < *r* < 350 μm) [22, 23].

Therefore, to investigate how individual fibrosarcoma cells within the spheroid contributed to the overall invasion rate into the surrounding 3D matrix, and to take into account the local cell density, spheroids grown for 3, 5, and 7 days were cryo-sectioned at a thickness of 10 μm and analyzed using quantitative fluorescence microscopy (see more details under Methods). Cells and their nuclei were visualized using DAPI staining to detect nuclear DNA and fluorescent labeling of the major cytoskeleton filamentous protein, F-actin (Figure 1D and 1E). Analysis of fluorescent images of the mid-plane sections of spheroids showed an exponential decay of the distribution of cells within the spheroid. Cell density was significantly higher at and near the geometric center than at the edges of the spheroids and steadily decreased along the radial axis (Figure 1F). We observed a 6-fold increase in the cell density near the geometric center, which is larger than the 2-fold increase previously reported for cohesively growing spheroids under confined environments [23, 24].

Mean-squared displacement (MSD) has been extensively used to characterize migratory behaviors of cells [10]. The cell density distributions in the spheroids also allow us to evaluate dissemination dynamics of fibrosarcoma cells using an approximate measure of the density-weighted ensemble-averaged mean squared displacement profile among all cells in spheroids at different time lags and also to compare directly with migratory profile of homogeneously distributed population of HT1080 cells in the same type of 3D matrix (Figure 1G and 1H). Analysis of this density-weighted ensemble-averaged MSD profile indicated that fibrosarcoma cells moved into the matrix with an exponent of α = 0.97 ± 0.13, suggesting that global cell invasion weighted by the local density of cells in the spheroids followed random-walk statistics (α = 1). Interestingly, cells within the spheroid showed a two-fold higher effective diffusivity constant when compared to a homogeneously distributed population of single cells (dispersed) embedded in the same type of 3D collagen matrices [10], indicating that cells in the spheroids were more motile than a population of homogeneously embedded cells in 3D gels at low density (Figure 1H).

### Spatio-temporal distributions of cell morphology within the spheroid

**Figure 2:**
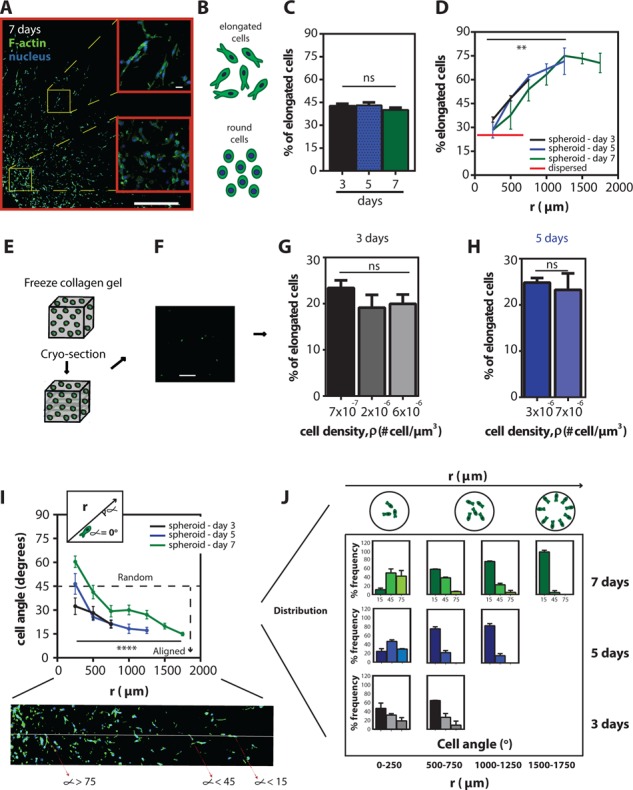
Shift in spatio-temporal cell morphology and cell alignment inside the multicellular spheroid **A.** Representative fluorescent micrograph of a mid-plane section of a spheroid embedded for 7 days showing the morphologic difference between cells close to the periphery and cells in the middle of the spheroid (blue-nuclei and green-actin filaments). Scale bar represents 500 μm and 20 μm in the inset. **B.** Schematic of elongated vs. round cells. **C.** Mean percentage of elongated cells ± SEM in the sections at 3, 5, and 7 days. **D.** Mean percentage of elongated cells ± SEM as a function of radius for 3, 5, and 7 days and for a population of homogeneously distributed single cells embedded inside 3D gels. **E.** Schematic of homogeneously distributed single cells inside collagen gels prior to cryo-sectioning. **F.** Representative fluorescent micrograph of a population of homogeneously distributed single cells in a collagen gel (EGFP-tagged). Scale bar, 200 μm. **G** and **H.** Mean percentage of elongated cells ± SEM as a function of local cell density for cells incubated in the gels for 3 and 5 days. **I.** Mean cell angle ± SEM as a function of radius for 3, 5 and 7 days. Inset shows the angle measurement and the fluorescent micrograph shows the cells' angle against a line through the origin of the aggregate. **J.** Angle frequency distribution as a function of radius and time, the schematic on top shows the spatial arrangement of the cells within the spheroids.

The highly invasive behavior of fibrosarcoma cells in the collagen matrix was observed by monitoring the propagating front of the spheroid. This analysis indicated that the rate of invasion was different depending on the location of cells within the spheroid when compared to the MSD results. Therefore, to explore how fibrosarcoma cells were spatially distributed inside the spheroids and to determine whether cells showed different migratory patterns within the spheroid, we examined the morphology of cells within the spheroid (Figure 2A). The morphology of HT1080 fibrosarcoma cells correlates strongly with their migratory potential in 3D matrices [25]. Based on the aspect ratio of the cell (denoted asp), we categorized cells as either elongated (asp > 1.05) or round (asp < 1.05) (Figure 2B); and the percentage of elongated *vs*. round cells as a function of time along the radial direction were measured (Figure 2C). Forty-five percent of cells were elongated inside the spheroids regardless of the time for which the spheroid had been embedded inside the 3D matrix (Figure 2C). However, there was a significantly higher percentage of elongated cells at the periphery of the spheroid: only ~30% of cells were elongated in the center of the spheroid, while ~70% were elongated at the periphery of the spheroid (Figure 2D). Moreover, the fraction of elongated cells inside the spheroid was significantly higher than for a population of individual cells homogeneously distributed (dispersed) at low density in a 3D matrix (Figure 2D). The percentage of elongated cells in homogeneously seeded cells in 3D matrices at different cell densities was ~ 25%, regardless of how many days the cells had been embedded in the matrices (Figure 2E–2H). Furthermore, we found that the cells in the spheroids were not only elongated, but also highly aligned in the radial direction. This alignment was measured from the angle between the long axis of the cell body and a line going through the origin of the spheroids (Figure 2I). This degree of cell alignment increased as a function of time (Figure 2J).

Together, these results demonstrate that the elongation and degree of alignment of fibrosarcoma cells are spatially dependent within the spheroid; both are significantly higher towards the periphery of the spheroid than at its core. In addition, the percentage of aligned cells was not only a function of space, but also a function of time.

### Cells have distinct motility profiles within tumor spheroids

**Figure 3:**
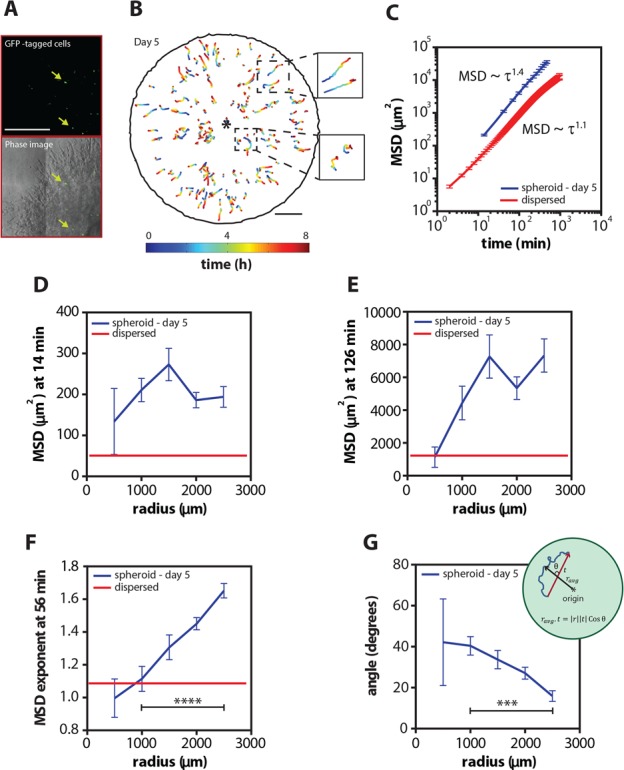
Cells move increasingly persistently as a function of radius inside the multicellular spheroid **A.** Representative micrograph of EGFP-tagged cells and overlay of phase and fluorescent images inside the multicellular spheroid. **B.** Cell trajectories as a function of time and radius of the EGFP cells tracked inside a spheroid on day 5. Insets to the right show zoom-ins of the trajectories in the center and the periphery of the spheroid. **C.** Mean square displacements (MSD) ± SEM as a function of time on day 5 and for a population of homogeneously distributed single cells (dispersed) embedded inside 3D gels. **D** and **E.** MSD ± SEM as a function of radius at time lag of 14 and 126 min on day 5 and for a population of homogeneously distributed single cells embedded inside 3D gels. **F.** MSD exponent at 56 min as a function of radius on day 5 and for homogeneously distributed single cells embedded inside 3D gels. **G.** Angle between trajectory axis and radial axis as a function of radius on day 5. Inset shows the angle measurement. Scale bars represent 500 μm and 1 mm for the cell trajectories. EGFP-labeled cells inside 7 different spheroids was analyzed and over 150 cells were tracked.

To discern whether these complex spatial and temporal morphological distributions within spheroids may be implicated in patterns of cell motility, we next studied single-cell motility inside the cell spheroids at single-cell resolution. To determine the motility patterns of individual cells within the spheroids, we formed spheroids composed of 10% EGFP-tagged and 90% wild type cells (and same overall density as used above). We tracked EGFP-tagged cells at different location within the spheroid for 8 h at 14-min time intervals (Figure 3A). We tracked cells starting at a radius of 500 μm because of the difficulty in tracking cells at the innermost radius. Therefore, in Figure 3, 500 μm does not represent the initial radius but only the radius at which we started tracking individual cells within the spheroids. We also checked for cell viability using the proliferative marker Ki67 (Supplementary Figure S5A–S5D): we only found a significant decrease in Ki67-positive cells in the core of the spheroids at day 7. This decrease is observed for radii < 500 μm, indicating that the tracked cells are not dead (Supplementary Figure S5A–S5D). Analysis of cell trajectories readily showed that cells close to the periphery of the spheroids moved persistently in the radial direction, as opposed to the cells at or close to the center of the spheroids (Figure 3B).

The ensemble-averaged MSD of individual cells in the spheroid displayed a power-law behavior, *MSD* ~ τ^α^ with an exponent *α =* 1.40 ± 0.04, suggesting that, on average, individual cells within the spheroid moved directly and not randomly (Figure 3C and Supplementary Figure S1A). Importantly, the motility patterns of individual cells at long time lags were strongly dependent on the location of the cells in the spheroid (Figure 3E and Supplementary Figure S1C). For example, cells displayed large MSDs when they were distant from the center of the spheroid suggesting that cells became more motile as they move toward the periphery of the spheroid (Figure 3E and Supplementary Figure S1C). In addition, cells at the periphery of the spheroid were more motile and more directed (Figure 3F and Supplementary Figure S1D and S1E), and they moved along a radial direction (Figure 3G and Supplementary Figure S1F–S1I), in contrast to cells near or at the spheroid center. The same trends were found for spheroids embedded in 3D matrices for 3 and 7 days (Supplementary Figure S1A–S1I). Furthermore, the cells were not only more directed as a function of radius but also as a function of time (Supplementary Figure S1G): cell speed in the radial direction significantly increased with time.

These results are consistent with our above estimation of cell motility based on spheroid invasion distance (Figure 1C). For comparison, we analyzed the motility of a population of homogeneously distributed single cells at low density inside 3D collagen matrices. As expected, individual cells well-dispersed in a 3D matrix moved randomly (*α* = 1.10 ± 0.01) and the MSD values were significantly smaller (Figure 3C–3F and Supplementary Figure S1B–S1E). It should be noted that our result shows a quantitative difference in MSD exponent between direct computation of MSD from cell tracking and MSD estimated from the cell density distribution in spheroid. This can be explained by the fact that cells in the core of the spheroids which account for the majority of the cell population are underrepresented in the cell tracking data due to difficulty in tracking cells at the core (Supplementary Figure S2C and S2D). In fact, our data shows that the MSD exponent for cells near the spheroid core (radius ~ 500 μm) has an exponent of approximately 1 (Figure 3F), in agreement with the approximation of density-weighted MSD profiles.

These results demonstrate that cells located at the spheroid periphery have a directed and persistent invasion within the surrounding matrix that is not observed when a population of cells is homogeneously distributed in a 3D matrix. Together, this data shows the spatio-temporal motility profile of cells within tumor spheroids: cells transition from random to a highly persistent invasion mode in the radial direction at the periphery of the spheroids.

### Collagen density modulates the rate of invasion but does not change the spatio-temporal distribution of cell morphology

**Figure 4:**
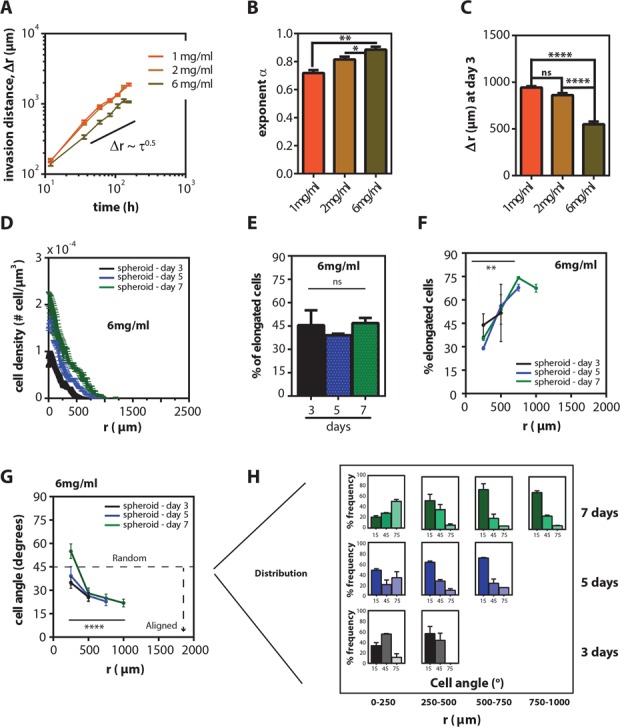
Collagen concentration modulates invasion distance, but does not change the spatio-temporal distribution of cell morphology **A.** Time-dependent mean invasion distance, *r(t)–r*(0) ± SEM of HT1080 spheroids at different collagen concentrations (1 mg/ml-orange, 2 mg/ml-yellow, 6 mg/ml-brown) **B.** Mean exponent of invasion ± SEM for different collagen concentrations. **C.** Mean invasion distance, Δr, ± SEM on day 3. **D.** Mean cell density ± SEM for spheroids as a function of radius measured from mid-plane cryo-sections on 3, 5, and 7 days inside 6 mg/ml gels. **E.** Mean percentage of elongated cells ± SEM in the sections at 3, 5 and 7 days inside 6 mg/ml gels. **F.** Mean percentage of elongated cells ± SEM as a function of radius on 3, 5, and 7 days inside 6 mg/ml gels. **G.** Mean cell angle ± SEM as a function of radius at 3, 5 and 7 days inside 6 mg/ml gels. **H.** Angle frequency distribution as a function of radius and time inside 6 mg/ml gels.

To identify potential mechanisms for the spatial effects on cell migration and to address whether the invasion of spheroids depended on matrix properties, we embedded spheroids in collagen matrices of different concentrations. Tumor spheroids embedded in both 1 mg/ml and 6 mg/ml collagen matrices invaded in a similar fashion; the exponent of invasion was *β* = 0.71 ± 0.05 and 0.86 ± 0.07 for 1-mg/ml and 6-mg/ml collagen matrices, respectively (Figure 4A and 4B). However, the spheroid invasion rate was modulated by the collagen density of the matrix: spheroids within a 1 mg/ml collagen matrix showed a 1.7 times longer invasion distance than within a 6 mg/ml collagen matrix (Figure 4C and Supplementary Figure S2B). Fluorescently stained cryo-sections of spheroids in 6 mg/ml collagen matrices revealed that, even for a low invasion rate, cell distribution and polarized morphological profiles were qualitatively similar to cells in spheroids inside 2 mg/ml collagen matrices (Figure 4D–4H). Together, these results indicate that collagen concentration regulates the rate of tumor spheroid invasion, but does not qualitatively change overall invasion patterns.

### Cells require contractility and integrin-based adhesion to persistently invade through collagen networks

To determine whether cell motility involve tension to reorganize the collagen matrix and direct the dissemination of cells, we visualized the real-time interaction between multicellular spheroids and collagen by fluorescently labeling the collagen [26]. This result revealed that cells within the spheroid reorganized collagen and redistributed it to its periphery within the first 7 h after spheroid embedding (movie S1). It was only after this enrichment of the collagen at the periphery of the spheroid that cells had the capability to invade.

**Figure 5:**
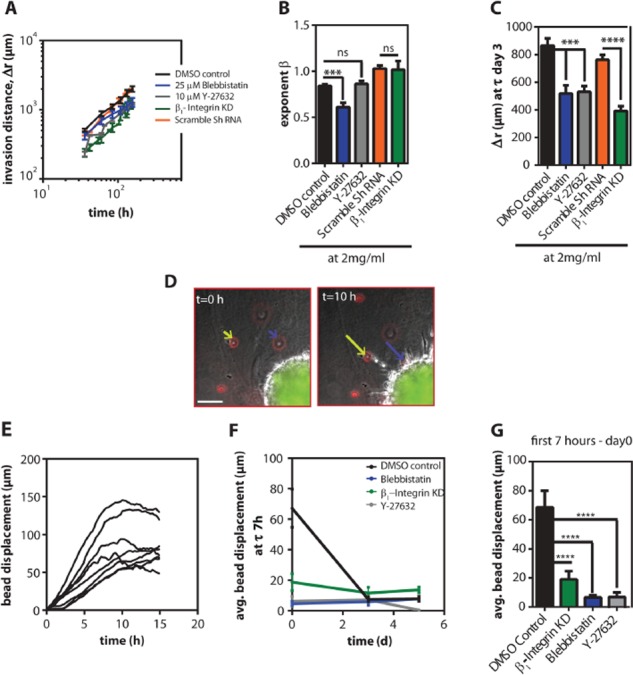
Cells required contractility to persistently invade inside 3D collagen gels **A.** Mean time-dependence of the invasion distance, *r(t)—r(0)*, ± SEM of HT1080 spheroids inside 2 mg/ml gels treated with 25 μM blebbistatin, 10 μM Y-27632 and the respective DMSO control and β_1_-integrin knock-down (KD) and the scramble shRNA control. **B.** The exponent of invasion ± SEM values for the different conditions. **C.** Mean invasion distance, Δr, ± SEM estimated on day 3. **D.** Typical micrographs showing the collagen probed with fluorescently labeled beads and their displacement as the cells start to invade into the collagen matrix. Scale bar, 100 μm. **E.** Single bead displacements as a function of time in the DMSO control. **F.** Average displacement ± SEM values as a function of time for the different conditions. **G.** Average bead displacement under the different pharmacological conditions for the first 7 h after the cells were embedded in the gel.

To understand whether cell contractility and cell-ECM interactions were required for the invasion of cell spheroids, we abrogated cell contractility by treating the spheroids with an inhibitor of Rho-associated protein kinase (ROCK) (Y-27632) and an inhibitor of non-muscle myosin II (blebbistatin). The invasion distance of spheroids was significantly reduced by blebbistatin and Y-27632. However, the invasion mode indicator (*β*) was only significantly reduced by treatment for blebbistatin to *β* = 0.61 ± 0.05 (Figure 5A–5C).

β_1_ integrin is a transmembrane adhesion molecule that mediates adhesion of HT1080 cells to collagen I. To determine whether cell-ECM interactions were required for invasion we depleted β_1_ integrin using shRNA. Depletion of β_1_ integrin significantly decreased the invasion distance, but not the invasion mode (Figure 5A–5C). Together, these results suggest that β_1_-mediated cell-matrix adhesive interactions and myosin II-mediated cell contractility both regulate the invasion distance, but only cell contractility is required for directed tumor spheroid invasion mode.

We next assessed the contractile forces exerted by the tumor spheroids within the 3D matrix by tracking fluorescently-labeled beads embedded in the matrices; the movement of beads indicate the magnitude and direction of traction forces applied by cells on their surroundings [27–29]. Within 7 h after spheroids were embedded in 3D matrix, the beads near the spheroid periphery collectively moved toward the spheroids (Figure 5D). On average, the displacement of beads for spheroids in the DMSO control group was ~ 68 μm in 7 h (Figure 5E–5G). However, when spheroids were treated with Y-27632 or blebbistatin, the direction of beads movement remained the same but the displacement was significantly reduced to 6 μm in the first 7 h (Figure 5F and 5G).

Global contractility of the matrix by spheroids also requires cell-matrix interactions. Spheroid containing β_1_-integrin-depleted cells showed significantly reduced collective contractility: the magnitude of bead displacements decreased to 19 μm in 7 h (Figure 5F and 5G). Interestingly, this contractility by the spheroids decayed with time. After 3 days in 3D collagen matrices, the collective movement of beads by the multicellular spheroids in DMSO control group was reduced by 9-fold and plateaued at 7 μm (Figure 5F).

Together, our results reveal the dynamic tension patterns exercised by spheroids inside 3D matrices and suggest that cellular contractility in spheroids induce collective traction forces, which ultimately result in a directed dissemination process at the periphery of tumor spheroids.

## DISCUSSION

In this work, we adopt complementary methods to study and characterize the dynamic dissemination of fibrosarcoma cells from tumor spheroids in 3D collagen matrices.

Our study reveals that cell dissemination from spheroids is a complex spatio-temporally dependent process. Cells adopt distinct migration patterns that critically depend on their radial positions within the spheroids and on time. Overall, cells show an increased degree of directed migration (measured by the MSD exponent) and elevated diffusivity as a function of the radial position within the spheroid and as a function of time. Thus, cells near the periphery of spheroids disseminate with faster rate toward the matrix, but they only account for < 10% of the cell population within the spheroid. This small population of cells is the one that governs the invasion distance of the spheroids and is different from the case of cohesively growing spheroids, which switch from exponential to linear growth after a necrotic core is developed [22, 23]. We observe a continuous linear invasion profile as a function of time even after a decay in the Ki67-positive cells in the core of the spheroids (Supplementary Figure S5A–S5D). We believe that this difference is due to the fact that, in our case, cells are highly invasive and the “spheroids growth” is mostly governed by the migration/invasion of the cells at the periphery, while in other studies [22, 23], it is cell division/proliferation that governs the rate of spheroid growth.

In addition, we also observed about a 6-fold increase of the cell density around the geometric mean when compared to the 2-fold increase observed in cohesively growing spheroids [22, 23]. In previous studies, the spheroids were grown in strongly confined environments and contact inhibition/cell volume could have been the limitation for a 2-fold increase in cell density. For instance, it was recently shown that a reduction in cell volume decreases cell proliferation in spheroids [30]. In our case, once HT1080 cells start invading through the collagen matrices they form a less compact spheroid, which may allow more space for cell division to occur; therefore, increasing the cell density. Additionally, even though this cell line is highly invasive, our single-cell motility analysis shows that the invasion profiles (i.e. motility magnitude toward radial direction of spheroid) are highly dependent on the cell's radial location within the spheroid. The cells closer to the core do not migrate effectively as the ones near the periphery; partly explaining the high cell density at the core of the spheroid.

Furthermore, our study shows that myosin-based contractility is critical to induce the directed dissemination of cells into the surrounding matrix. This directed cell dissemination is shared with breast cancer MDA-MB-231 spheroid model (Supplementary Figure S3B). Previous studies aimed at mathematically modeling the motility of cells at the invasive front of U87 glioblastoma tumor spheroids have included either a significant radial velocity bias [31] or an internal polarity at the cell invasion front [32]. Together, these results suggest that directed cell dissemination is a general process for cells at the periphery of spheroids in 3D collagen matrices.

We found that major reorganization of the collagen matrix occurs within the first 7 h after spheroid embedding and this is likely due to the collective cell traction force as indicated from the displacements of beads embedded in the 3D matrix. From previous work in our lab [17], we find that individual HT1080 cells can displace beads for approximately 4.5 μm (in random orientation) in 1.5 h inside 3D collagen matrices of 2 mg/ml. For HT1080 spheroids inside 2 mg/ml gels, we found that the beads move persistently toward the spheroid at a rate of approximately 68 μm in 7 h (i.e 9.7 μm per hour). Therefore, the overall magnitude of contractile forces generated by the spheroids is at least 3-fold higher than for a population of homogenously distributed cells. The collagen reorganization and subsequent invasion may be a result of several potential factors, such as hypoxia, chemotaxis, collagen alignment and/or physical proximity. For instance, it has been shown that hypoxia regulates ECM remodeling and promotes invasion and metastasis [12].

Previous studies have shown that perpendicular fiber alignment correlates well with poor prognosis in breast cancer [13, 33]. Further, population of homogeneously distributed single cells invading in 3D collagen gels have been shown to migrate persistently on aligned collagen [34]. These results suggest that one of the key factors in mediating this directed dissemination process for cells at the spheroid-ECM interface is the alignment of collagen fibers. When this collagen reorganization is abrogated by pharmacological treatment using both blebbistatin and Y-27632 treatments, which target contractility, there is a decrease in the invasion distance of the spheroids.

Generating cryo-sections of the tumor spheroids allowed for the analysis of individual cells at single-cell resolution across the entire spheroid. This analysis showed that there is a significantly higher percentage of elongated cells at the periphery when compared to the center of the spheroid (70% *vs*. 30%) and that these cells are highly aligned in the radial direction. It has been previously shown that population of homogeneously embedded single cells migrating through collagen matrices or plated on tumor sections rich in collagen fibers exhibit an elongated morphology [7, 25, 35]; and that upon β_1_ integrin blocking with mAb 4B4, there is a loss in cell elongation and polarization [7]. In the current study, cells transition in morphology from round to elongated as a function of increasing radial position. The fact that most of the round cells are in the middle of the cluster could be explained by cells adopting MMP-independent amoeboid migration [18] because the leading or elongated cells presumably degrade the collagen creating tracks for other cells to follow with less resistance; therefore, allowing for the apparent MMP-independent migration. Furthermore, from our morphology analysis we found that the polarity of cell morphology and its alignment to the radial axis of the spheroid is highly correlated with the degree of the directed motion. This result suggests that the analysis of cell morphology within spheroids could potentially be developed into a powerful and high-throughput platform for cancer cell screening for drug discovery and drug response.

## METHODS

### Cell culture and cell spheroids

HT1080 cells (ATCC, Manassas, VA) were cultured in Dulbecco's modified Eagle's medium (DMEM, Mediatech, Manassas, VA) supplemented with 10% (v/v) fetal bovine serum (FBS, HyClone, Logan, Utah) and 0.1% gentimicin (Sigma, Saint Louis, Missouri). Cells were maintained in a humidified environment at 37°C and 5% CO_2_ during culture and live cell imaging. Cells were passaged every 2–3 days for a maximum of 20 passages. HT-1080 cell spheroids were formed in non-adhesive round-bottom 96-well plates following a protocol modified from [36]. Briefly, monolayers of fluorescently labeled HT1080 cells were trypsinized, suspended in spheroid formation medium (3:1, DMEM:methocult H4100 (Stemcell techonologies, Vancouver, BC)) and diluted to a density of ∼1 × 10^6^ cells/ml; 100 μl of this diluted cell solution was seeded into each well of the 96-well plate. Cells were then centrifuged in the well-plate at 1200 RPM for 7 min; the 96-well plate was rotated and centrifuged again to guarantee roundness. Cells were incubated under standard conditions (as mentioned above) for 2 days.

### Lentiviral production and transduction

Lentiviral vector of EGFP (pCS-CG) was purchased from Addgene (Cambridge, MA). The second-generation lentivirus was produced as described previously [37]. Briefly, 293T cells (ATCC) were transiently co-transfected with three plasmids, including lentiviral vector DR 8.91 and pMDG-VSVG, using the standard calcium phosphate precipitation method. After a 22–24 h transfection, the medium was replaced with fresh medium. Lentiviral particles were harvested 24 h later by collecting the medium and filtering it through a 0.45 μm filter (Millipore, Bedford, MA) to remove cell debris. The filtered medium was then stored at −80°C. Cells were transduced several times with lentivirus containing 8-μg/ml polybrene. Fluorescence-activated cell sorting (FACS) was then used to obtain the top 10% of EGFP expressing cells.

### β_1_-integrin depletion

The online program Dharmacon (http://www.dharmacon.com) was used to design the RNAi sequences targeting mRNA of β_1_-integrin. The sequence used was TGCCTACTTCTGCACGATGT (174). Western blotting was performed and analyzed using ImageJ (NIH) to confirm the successful depletion of the protein (above 90% efficiency).

### 3D type-I collagen matrices

Collagen matrices were prepared as previously described [7] by mixing culture medium and 10x reconstitution buffer, 1:1 (v/v), with soluble rat tail type I collagen in acetic acid (BD Biosciences, San Jose, CA) to achieve the desired final collagen concentrations of 1, 2, and 6 mg/ml. 1 M NaOH was then added to normalize the pH (pH 7.0, 0.023*volume collagen = volume of 1 M NaOH as directed in the BD protocol). All reagents were kept chilled in an ice bath, and care was taken to avoid bubble formation. For homogenously distributed single cell experiments, 500 μl of collagen mixture at cell density of 3.6 × 10^4^ cell/ml was placed in a 24-multiwell culture plate (Corning, Tewksbury, MA). Plates were incubated under standard culture conditions overnight. Medium was added to the gels 2 h prior to live imaging. For cell spheroids, 600 μl of collagen solution was added to 8 mm ID square cells (Vitrocom, Mountain Lakes, NJ). To place the spheroid in the middle of the square cell, spheroids were slowly aspirated with a 1 ml pipette tip from the 96-well plate and placed on the lid of a 10 mm petri-dish, the spheroid formation medium was discarded and the spheroid was aspirated into a 200 μl pipette tip with 20 μl of collagen solution. Once in the pipette tip, the spheroid was placed in the middle of the square cells/glass cuvettes to guarantee a 3D microenvironment. The cuvettes were incubated under standard conditions for 30 min before adding 300 μl of warm culture medium on top of the gel. For experiment with fluorescently labeled beads (Molecular probes by Life Technologies, REFF8834, Frederick, MD), 10 μm beads were added to the gel mixture for a final bead concentration of 7.2 × 10^4^ beads/ml.

### TAMRA-collagen I labeling

Rat tail collagen I was labeled as previously described [26]. Briefly, tetramethylrhodamine (TAMRA) azide (Life Technologies, Grand Island, NY) was dissolved in DMSO to a final concentration of 10 mg/ml. Then, 1 ml of high concentrated rat tail collagen I was injected into a presoaked 10,000 MWCO dialysis cassette (Life Technologies) and dialyzed overnight against 1 L of labeling buffer (0.25 M NaHCO_3_, 0.4 M NaCl). After the dialysis, the collagen was mixed with 1 ml of TAMRA solution (100 μl of the 10 mg/ml TAMRA solution diluted in 900 μl of labelling buffer). This collagen/TAMRA solution was then incubated overnight with rotation at 4°C and then dialyzed the next night against 1 L of labeling buffer to remove excess dye. Subsequently, this solution was again dialyzed overnight in 1 L of 0.2% (v/v) acetic acid solution, pH 4. The final concentration of TAMRA-labeled collagen was calculated from the measured final volume, and the initial volume and collagen concentration.

### Cell spheroid growth

The mid-planes of cell spheroids embedded in 3D collagen matrices were imaged at low magnification (10x) once a day for 7 days with a Nikon TE2000 microscope. The cell spheroid area was obtained using Nikon elements software by manually tracing the cell spheroid periphery as depicted in Figure 1B.

### Drug treatments

Specific myosin II inhibitor blebbistatin and ROCK inhibitor Y-37632 (Sigma) were dissolved in DMSO and added to the medium on top of the collagen gel for a final concentration of 25 μM and 10 μM respectively. The warm medium containing the drug was added right after the formation of the collagen-embedded spheroid complex.

### Live-cell tracking of individual cells within the spheroids and inside collagen gels

Multicellular spheroids containing 10% of fluorescently labeled EGFP-HT1080 cells were imaged at low magnification (10X) for 8 h every 14 min in the mid-plane of the spheroid with a Nikon swept field microscope. For analysis of homogeneously distributed cells in collagen gels, cells were imaged for 25 h every 2 min at a 1000 μm above the bottom of the 24-well plate at low magnification (10x) with a Nikon TE2000 microscope. Measurements were performed by tracking single cells using Metamorph image recognition software (Molecular devices, Sunnyvale, CA).

### Mean squared displacement (MSD) of individual cells homogenously distributed in the matrix

Cells were distributed in collagen matrices at low density. The mean square displacement (MSD) was calculated as previously described [25]. A custom MATLAB program (MathWorks, Natick, MA, USA) was used to calculate the MSD using x, y coordinates obtained from the cell tracking using the equation:

$$\text{MSD }=\;\langle {\left[x\left(\left(t+\Delta t\right)\right)-x\left(t\right)\right]}^{2}+{\left[y\left(t+\Delta t\right)-y\left(t\right)\right]}^{2}\rangle$$ (1)

Note that x and y coordinates are the 2D projection of 3D cellular movements with the assumption that cell movements were isotropic as previously described in [25]. Velocity measurements were performed by calculating the radial and tangential components of the trajectory vector of each cell with respect to the origin of the spheroid.

### Embedding collagen gels for cryostat sectioning

A previously described protocol to freeze glioblastoma spheroids [38] was modified. Briefly, at the desired time point, collagen gels were fixed in 4% paraformaldehyde (Electron Microscopy Sciences, Hatfield, PA) in PBS (v/v) overnight at 4°C. The collagen gels were gently detached from glass squared cells and submerged in 7 ml of 30% sucrose (Sigma) in 10X PBS and stored at 4°C for approximately 24 h. The gels were then submerged in a mixture of 30% sucrose and OCT (8:2 sucrose to OCT for 2 mg/ml and 7:3 ratio for 6 mg/ml) and frozen down on a dry ice/ethanol bath.

### Immunofluorescence microscopy

Cryostat slices were stained as previously described [39]. Briefly, the slices were rinsed with 1X PBS, blocked with 2% BSA (Sigma) in PBS for 30 min at room temperature and then incubated with anti-Ki67 antibody (ab16667) (Abcam, Cambridge, UK) at a 1:200 dilution overnight and then with fluorescent secondary at a 1:250 dilution and with Alexa-Fluor phalloidin 488 (Life technologies, Grand Island, NY) at a 1:40 dilution for 1 h. In the final step, DAPI ProLong ^®^Gold antifade reagent (Life Technologies) was added for nucleus staining. Stains were visualized using a Nikon TE2000 microscope with a Luca-R EMCCD camera (Andor Technology, South Windsor, CT).

### Computational estimation of the cell density inside the spheroid

The average radial local density of the cells was extracted by creating a histogram of the number of cells over a mesh of voxels defined on the equatorial slice cryostat image. Spherical symmetry was assumed. The number of cells was counted using the image segmentation tools in Matlab as illustrated in Supplementary Figure S4A. Each voxel had a fixed volume *V* that was chosen to be 2 × 10^4^*μm*^3^. Different values for the fixed volume did not affect the cell density profiles. The corresponding radial position *r* of each voxel was found by optimization given the local geometric constraints: cryostat slice thickness (10- *μm*), voxel volume and the tangential angle. The average cell density profiles *ρ*(*r*) were then obtained by averaging 20 voxel samples on each tangential direction for each *r* position. An estimate for the total number of cells is then given by:

$$N\;=\;4\pi \int_{0}^{R_{w}}\rho \left(r\right)r^{2}dr$$ (2)

where *R_W_* is the radius of the well in which the collagen-embedded spheroids were grown.

### Statistical analysis

Mean values, standard error of the mean (SEM), and statistical analysis were calculated and plotted using Graphpad Prism (Graphpad Software). Where appropriate, the following statistical analyses were conducted to compare means: two-tailed unpaired *t*-tests and one-way ANOVA analyses with Tukey post-test. In all data shown, ****, ***, **, *, and ns indicate *p* value < 0.0001, < 0.001, < 0.01, < 0.05, and > 0.05, respectively. α = 0.05 was used for all significance.

## SUPPLEMENTARY FIGURES


