# Supplementary material for: Collective cancer cell invasion induced by coordinated contractile stresses
Source: Oncotarget. 2015 Oct 30;6(41):43438–51. doi: 10.18632/oncotarget.5874 (PMC4791242; doi:10.18632/oncotarget.5874)
Supplement: Supplementary file 1 [file oncotarget-06-43438-s001.pdf]

SUPPLEMENTARY FIGURES

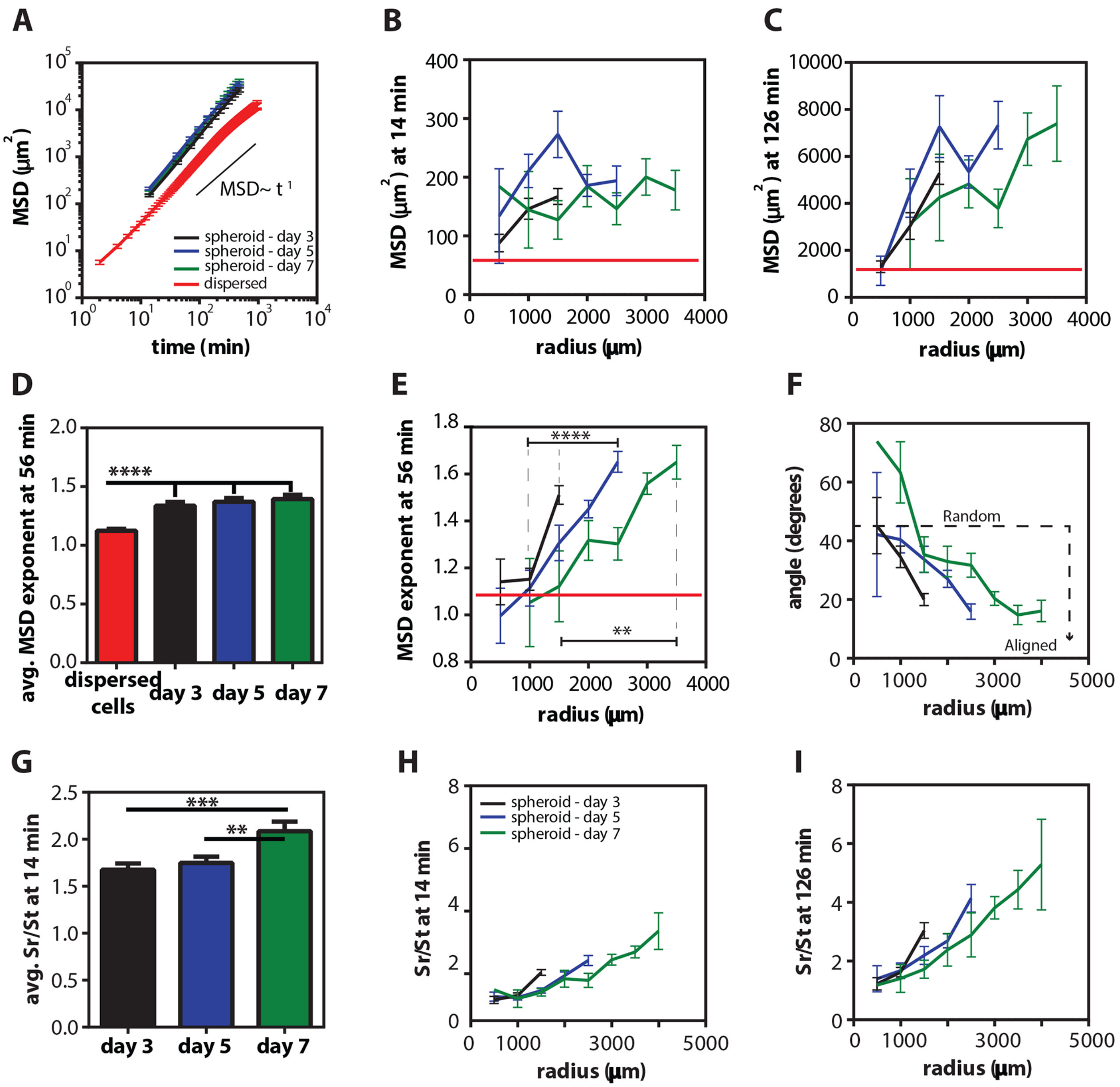

Supplementary Figure S1: Persistent movement of cells inside spheroids. A. Mean square displacements (MSD) ± SEM as a function of time for population of homogeneously distributed single cells (dispersed) and for individual cells inside spheroids grown for 3, 5 and 7 days. **B** and **C.** MSD ± SEM as a function of radius at time lag of 14 and 126 min for cells inside spheroids grown for 3, 5 and 7 days and for a population of homogeneously distributed single cells embedded in collagen gels. **D.** Average MSD exponent ± SEM at 56 min for cells inside spheroids on day 3, 5, and 7 compared to the exponent for homogeneously distributed single cells embedded inside the collagen gels. **E.** MSD exponent ± SEM at 56 min for homogeneously distributed single cells and for individual cells in spheroids as a function of radius on 3, 5 and 7 days. **F.** Angle between trajectory axis and radial axis ± SEM as a function of radius for spheroids on 3, 5 and 7 days. **G.** Average of the ratio between radial and tangential speeds (Sr/St) ± SEM at 3, 5, and 7 days. **H** and **I.** Mean Sr/St ± SEM at times 14 and 126 min as a function of radius on days 3, 5 and 7. EGFP-labeled cells inside 7 different spheroids were analyzed and over 149 cells were tracked per condition.

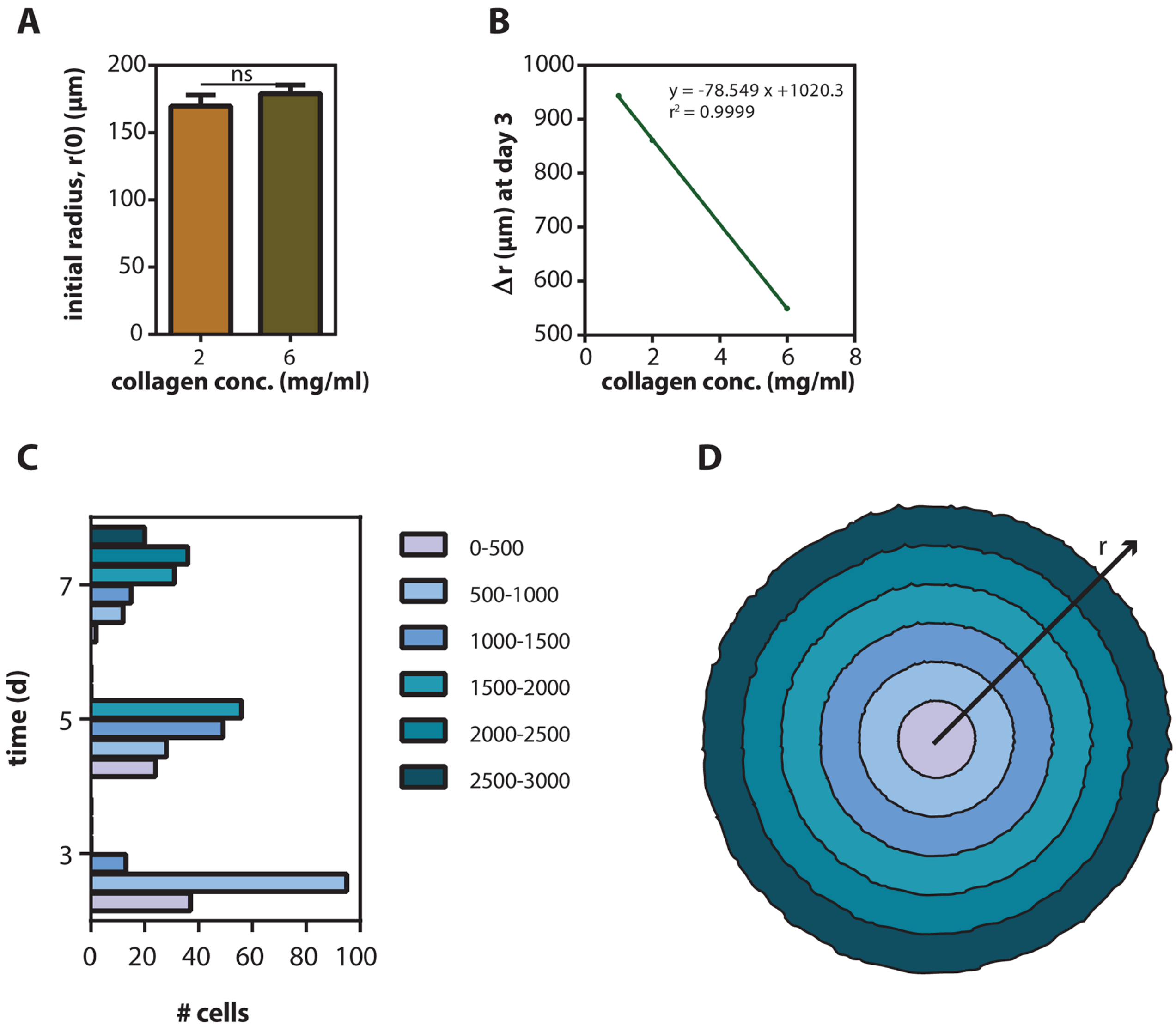

Supplementary Figure S2: Sample cell distribution of single cell motility within the spheroid. A. Mean initial radius ± SEM of spheroids as a function of collagen concentration. **B.** Avg. invasion distance at day 3 as a function of collagen concentration. **C.** Distribution of the number of cells analyzed within the different radii bin at 3, 5 and 7 days. **D.** Schematic of the radii bin sampled on B.

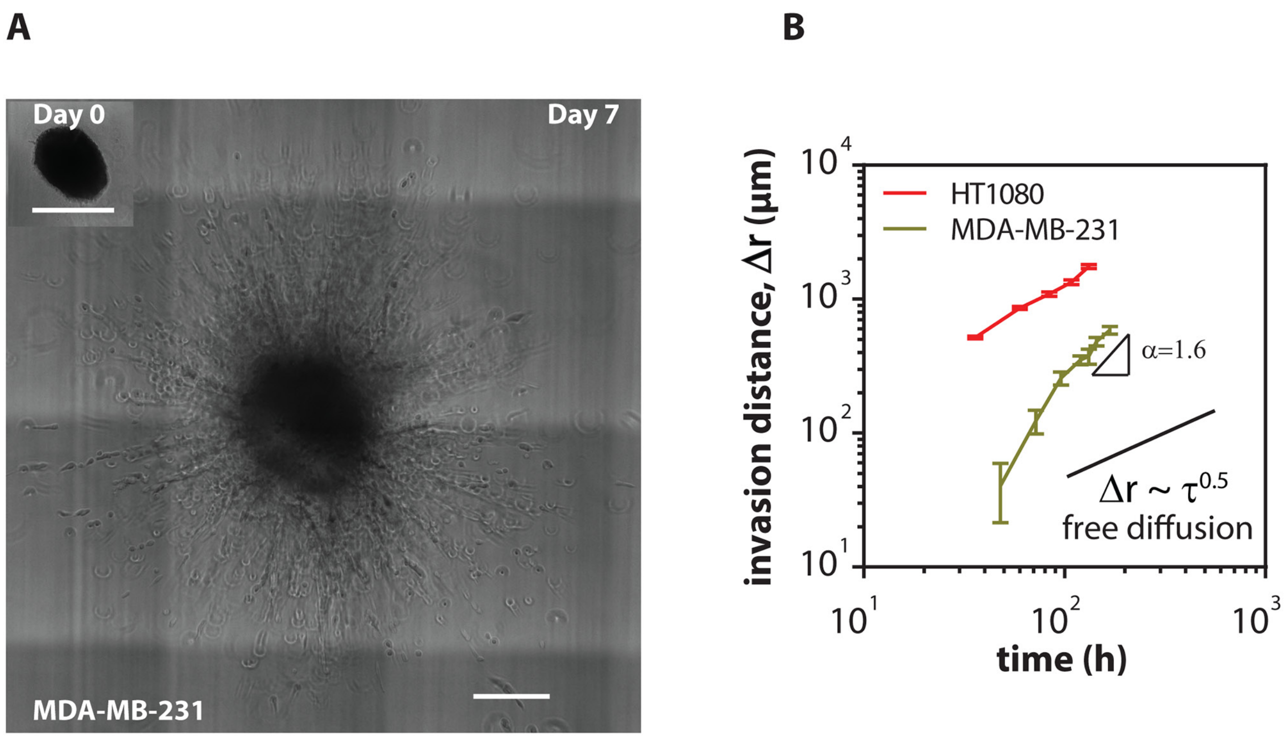

Supplementary Figure S3: MDA-MD-231 spheroids display directed dissemination inside 3D collagen matrices. **A.** Representative stitched phase-contrast images showing the evolution of breast cancer MDA-MB-231 spheroids at 0, and 7 days after embedding inside 2 mg/ml collagen matrices. **B.** Time-dependent mean ± SEM values of the invasion distance, $r(t) - r(0)$, of HT1080 and MDA-MB-231 spheroids inside 2 mg/ml gels. Inset shows the exponent of invasion for MDA-MB-231 cell spheroids. Scale bar, 500 µm.

binary image, obtained by thresholding

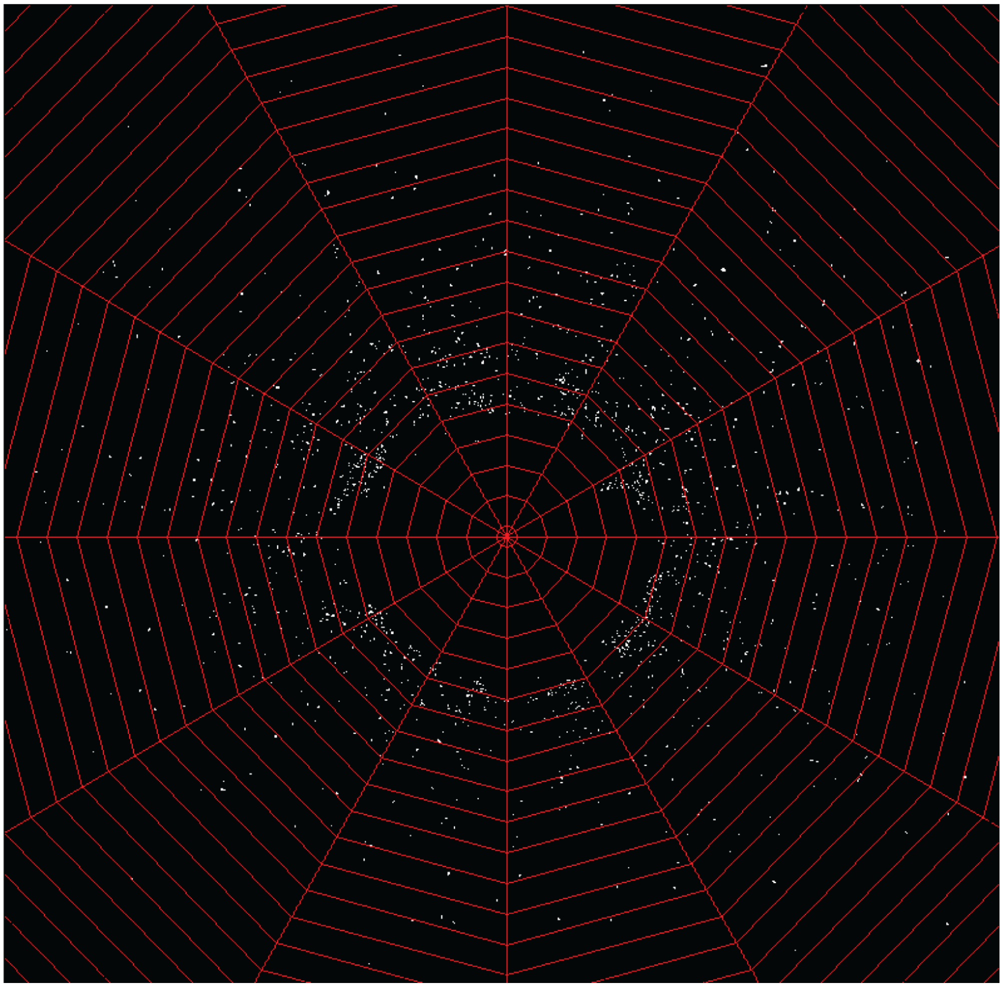

Supplementary Figure S4: Volume mesh on the equatorial plane slice for cell density measurements. Representative image of the volume mesh (voxels) created on cryo-section slides to measure cell density as a function of both radius and time.

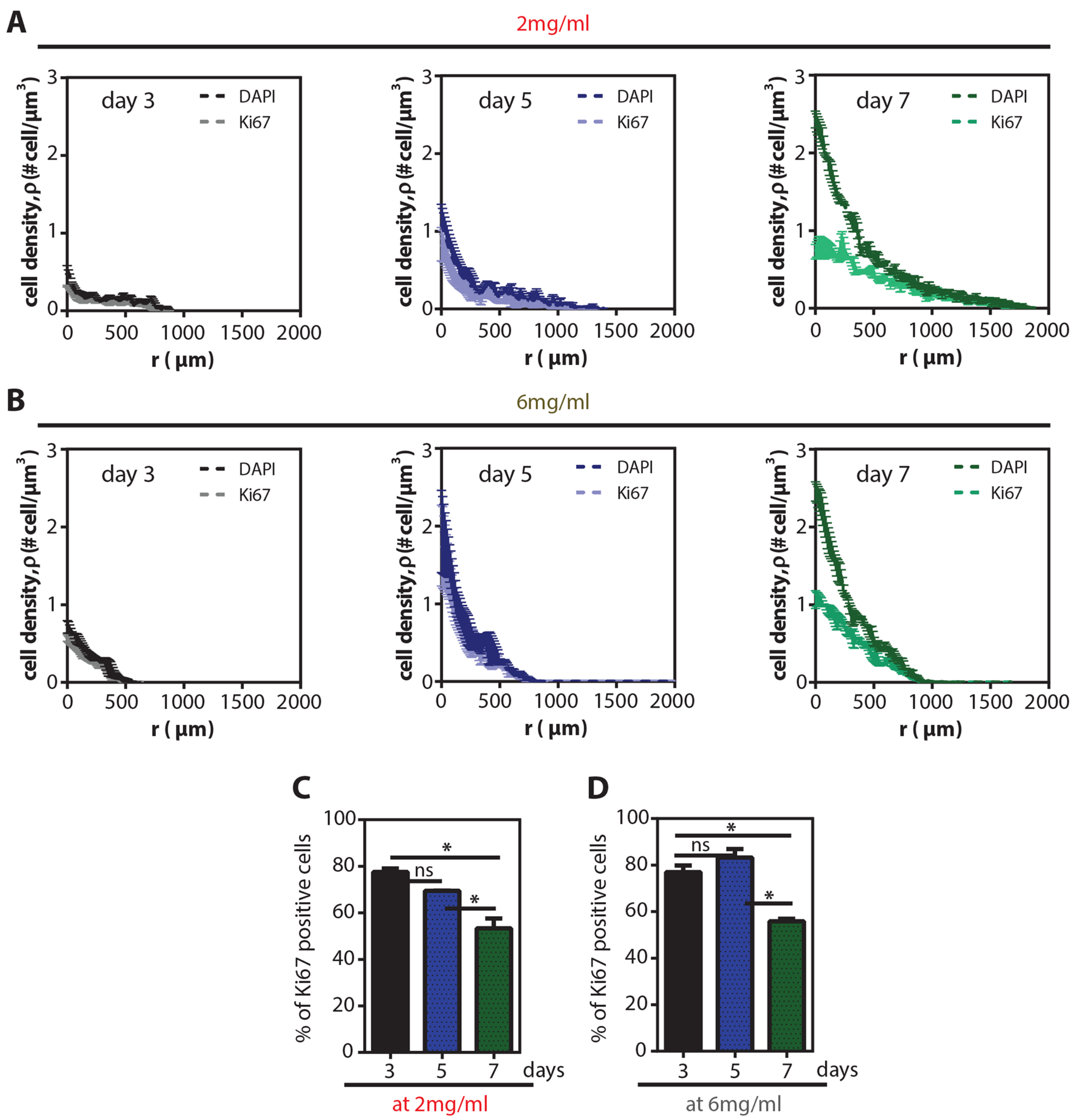

Supplementary Figure S5: The percentage of Ki67 positive cells decreases in the core of the multicellular aggregate as a function of time. A. Cell density ± SEM values as a function of radius showing nucleus (DAPI) and Ki67 stains measured from the mid-plane cryo-sections at 3, 5, and 7 days for multicellular aggregates grown inside collagen gels at 2 mg/ml. **B.** Cell density ± SEM values as a function of radius showing nucleus (DAPI) and Ki67 stains measured from the mid-plane cryo-sections at 3, 5, and 7 days for multicellular aggregates grown inside collagen gels at 6 mg/ml. **C.** Percentage of cells positive for Ki67 as a function of time for spheroids grown inside gels at 2 mg/ml. **D.** Percentage of cells positive for Ki67 as a function of time for spheroids grown inside gels at 6 mg/ml.
